# Understanding the Methodological Issues and Solutions in the Research Design of Stroke Caregiving Technology

**DOI:** 10.3389/fpubh.2021.647249

**Published:** 2021-04-16

**Authors:** Elton H. Lobo, Anne Frølich, Lene J. Rasmussen, Patricia M. Livingston, John Grundy, Mohamed Abdelrazek, Finn Kensing

**Affiliations:** ^1^School of Information Technology, Faculty of Science, Engineering and Built Environment, Deakin University, Geelong, VIC, Australia; ^2^Section of General Practice, Department of Public Health, University of Copenhagen, Copenhagen, Denmark; ^3^Innovation and Research Centre for Multimorbidity, Slagelse Hospital, Region Zealand, Denmark; ^4^Department of Cellular and Molecular Medicine, University of Copenhagen, Copenhagen, Denmark; ^5^Center for Healthy Aging, University of Copenhagen, Copenhagen, Denmark; ^6^Faculty of Health, Deakin University, Geelong, VIC, Australia; ^7^Faculty of Information Technology, Monash University, Clayton, VIC, Australia; ^8^Department of Computer Science, University of Copenhagen, Copenhagen, Denmark

**Keywords:** stroke, caregiver, design methodology, technology, issues, solutions, user-centered design

## Abstract

The rise in the number of cases of stroke has resulted in a significant burden on the healthcare system. As a result, the majority of care for the person living with stroke occurs within the community, resulting in caregivers being a central and challenged agent in care. To better support caregivers during the recovery trajectory poststroke, we investigated the role of health technologies to promote education and offer various kinds of support. However, the introduction of any new technology comes with challenges due to the growing need for more user-centric systems. The integration of user-centric systems in stroke caregiving has the potential to ensure long-term acceptance, success, and engagement with the technology, thereby ensuring better care for the person living with stroke. We first briefly characterize the affordances of available technologies for stroke caregiving. We then discuss key methodological issues related to the acceptance to such technologies. Finally, we suggest user-centered design strategies for mitigating such challenges.

## Introduction

The increase in healthcare costs has resulted in the transition of stroke care from inpatient to community-based services (1). As a result, caregivers are expected to take on the responsibility to provide essential support to people living with stroke (2). However, the lack of coordinated postdischarge care leaves the caregiver and the person living with stroke to often feel abandoned and unsupported (3). This leads to an increase in burden or strain on the caregiver (4). Research highlights that the burden of caregiving is a multidimensional concept that includes several adverse effects on the physical, psychological, social, and financial functioning of the caregiver (5). Because of this, the caregiver is at risk of impaired health, suboptimal cognitive functioning, poor mental health, disruption in household roles, reduced quality of life, and changes to important life goals and future plans (6). Therefore, there is a need to transform care support, increase access, improve quality of care, and reduce cost of care throughout the disease trajectory.

Recent advances in health information technologies have been gaining interest in supporting caregivers in stroke as they utilize a combination of information and communication technologies to provide a more practical, affordable, and user-friendly solution (7). Such technological solutions are unrestricted by the place or time and focus on empowering its user to improve participation, decision making, and commitment to treatment, thus improving overall health outcomes (8).

While health information technology solutions have the potential to better support caregivers, the process of providing such care within the community has proven to be a challenge (9) that needs to be considered in the design of any care support system. These challenges need to be addressed to ensure new technological solutions are acceptable to their target end users. However, to date, researchers do not fully understand the scope and complexity of including users in the design of the care support system (10). This leads to issues in adoption of the solutions proposed.

Our objective is to better inform future researchers on means to address this issue. To achieve this objective, we first characterize the potential of health technologies in stroke caregiving and issues faced by the user in accessing and using these technologies. We then review the methodological practices implemented to design these technologies. In doing so, we highlight the key methodological issues reported in the design of stroke caregiving technology. We also discuss various concerns addressed by the researchers during the development of such technological systems. Finally, we suggest user-centered design strategies that have proven instrumental for mitigating such challenges in the healthcare domain.

## Health Technologies in Stroke Caregiving

Health technologies in stroke caregiving consist of different means to promote interaction using web (11–13), Information and Communication Technology (ICT) (14, 15), mHealth (16–24), and telehealth systems (22). Web-based systems are those that are delivered through a browser on different devices such as computer, television, or mobile, with the requirement for access to the internet to use the service (11–13). ICT systems rely on a communication technology to connect numerous different devices (14, 15), mHealth relies on mobile devices (16–24), and telehealth relies on telecommunication devices such as telephone to promote interaction and support (22). The intention of these systems is to promote healthcare delivery and exchange over a wider geographic distance, thereby ensuring more effective and efficient care to the person living with stroke (11–24).

Technologies for stroke caregiving highlight the potential of these interventions in providing the caregiver with education, communication, monitoring, and rehabilitation support tools to promote better care for the person living with stroke (11–24). These technologies are designed to address specific needs of the caregiver identified through the use of surveys (17–19), interview (14–18), focus groups (11, 14, 16, 17), observations (14, 15, 18), and/or best practices from evidence-based literature (12, 13, 20–24). Caregivers adopting these technologies are satisfied with the ability to use them at any given place or time, while being able to interact and share information with people having similar experiences (13, 17, 18). Moreover, they allow for the caregiver to be reassured about their practices and techniques during recovery (17). Overall, the literature reports that the technologies employed to date have been effective (11, 18, 19) and acceptable (13, 17–19) in helping to support and manage the person living with stroke.

## Methodological Issues in Stroke Caregiving Technology

Technology for stroke caregiving is a useful tool to improve efficiency and quality of rehabilitation care (25). Despite widespread agreement of the potential of stroke caregiving technology in care and recovery, several researchers rely mostly on evidence-based approaches for the design of stroke caregiving technology (12, 13, 20–24). The aim of such processes is to ensure conscientious, explicit, and judicious use of best evidence guidelines created by credible research or best-practice guidelines or through systematic reviews and/or meta-analysis to make critical decisions regarding the design of the system (26). While theoretical models can form a solid foundation in the design of new technologies, the lack of understanding and ability to provide direct attention to the user suggests that it may be less effective for people with different chronic conditions (27). Furthermore, there are numerous concerns regarding the level of use by stroke caregivers. As a result, several research-based stroke caregiving technologies are not yet fully realized in commercial markets for use by caregivers of those with stroke (28).

The lack of realization of stroke caregiving technology in the market raises concerns around the methods and evaluation procedures in their design. These are exacerbated by the structure and design of the system and means by which the user interacts with it (29). Issues surrounding the structure, design, and user interaction could be better addressed through a detailed understanding of the user capabilities. These need to be acquired from user responses that cannot be determined through evidence-based theories (29). Moreover, only a few studies focus on understanding the range of factors associated with the interaction of the user and the system in stroke caregiving technology literature (13, 14, 17, 18, 23, 30) and more focus on its ability to meet the caregivers' needs in recovery (13–18, 20–23, 31–35).

While stroke caregiving technology should be designed to support the caregivers' needs during recovery, it must also consider the range of factors toward implementing necessary functionalities. This is because it could create risk for not only the caregiver but also the patients and medical professionals (36). For example, providing general information regarding the disease and not specific information related to the patient's condition could impact the quality of care. Therefore, the system implemented needs to account for an easy-to-use design, while also ensuring the data presented to the user are effective, easy to comprehend, and free from errors (37). Moreover, information provided to the user should be based on their specific needs (38), thereby limiting any confusion during care and recovery. These factors are similar to the studies by Cameron et al. (2), Creasy et al. (39), and Krieger et al. (40), which all suggest the need of caregivers to have personalized information that are easy to comprehend (2, 40–42) and are delivered at appropriate times (2, 42–45). While technology has the potential to provide personalized information and support (such as medication delivery, self-monitoring, and so on) through the use of context-aware systems (46); it has not yet been realized for stroke caregiving technology. Hence, it is clear that there is a lack of understanding of the available technology and user needs, which results in issues during the design and implementation of such technologies.

## User-Centered Design to Improve Stroke Caregiving Technology

The limitations of current and future stroke caregiving technologies can be reduced by better promoting user involvement in its design, development, and implementation (25). One such approach is user-centered design (47). The concept of user-centered design offers tangible, scalable, and reproducible methods to include relevant users in the healthcare process (48). Through the better inclusion of target end users during development, the developers can focus on observing and understanding the planning of care and recovery trajectories and tailor the technology to support the needs of the user during this process. This extends beyond traditional practices that tend to rely on evidence-based literature (13, 21–24) to develop and implement technologies to support stroke caregivers. However, it is important to note that the practices involved in user-centered design are not new to healthcare. For example, the development of medicines undergoes several modifications including understanding its effects and impact on the user prior to making it public and ensuring adoption. None of these medications is developed entirely based on evidence-based literature or personal experiences. This is similar to what user-centered design aims to achieve, but with technology solutions.

While some studies (13, 14, 17, 18, 23, 30, 35) have considered iterative user-centered design approaches and participatory design (PD) practices, the extent of implementation of these methods has not been fully described in the literature. For example, Sureshkumar et al. (18) focused on a user-centered design methodology for the design of an educational-based mobile application to support stroke caregivers; however, there is no explanation in the study of how they investigate users' needs and capabilities. Such knowledge could be used to conclude that educational support was the only need of stroke caregivers and that they are comfortable using a mobile-based application. These assumptions (i.e., education support delivered through mobile) are not always the case as highlighted in the studies involving the needs assessment (49) and technological capabilities (47) of caregivers in stroke. Similar assumptions were implemented in other user-centered design studies (14, 17, 23, 31), where the full breadth of caregivers' needs and capabilities were not investigated prior to designing the intervention. This led to issues in the initial design of the technology and a lack of integration of technology in the normal care practices during recovery as discussed in the previous section.

The lack of proper implementation of user-centered design practices could be due to three challenges: (i) understanding users' practices and needs, (ii) the codesign of innovative and sustainable solutions, and (iii) the technical and organizational implementation. These challenges were identified based on literature findings (50–53). This is important, as the success of any user-centered design study is dependent on a genuine user participation (54). Through the inclusion of users, it is possible to generate new insights and ideas that can embrace ambiguity and provide structured systematic innovation in public health. The healthcare literature has also highlighted the importance of user-centered design and the role of users in developing sustainable systems by creating actionable strategies to test, refine, and integrate the solution in the individual's daily activities (55). Hence, there is a need to suggest mitigation strategies in terms of guiding principles and tools and techniques that can support stroke caregivers, as shown in Table 1. These recommendations are based on our own and colleagues' experiences, some of which are gained in healthcare projects conducted to support different chronic conditions. However, these studies do not account for caregivers and would need to be studied further to gain greater insights to better support these individuals.

**Table 1:** Challenges, guiding principles, and tools and techniques in implementing user-centered design.

**Strategy**	**Guiding principles**	**Primary tools and techniques**
Developing a proper understanding of a diverse set of groups of users' practices and needs	Participatory design (PD) literature suggests genuine user participation (56) and getting firsthand experience with current work practices (57). Computer-supported cooperative work (CSCW) literature recommends, e.g., aligning concerns, focus on needs for awareness (10, 52), and being cautious expecting one group to deliver valuable data without getting valuable feedback (58)	Ethnographically inspired fieldwork: interviews, observations, workshops, thinking aloud, and so on
Codesign of innovative and sustainable solutions	PD literature recommend concurrent design of coherent visions for change (information technology systems, work organization, and mapping out the qualifications needed) and that special attention is given to anchoring visions with users, managers, and those responsible for the technical and organizational implementation (56)	Iterations of workshops, scenarios, and prototyping
Technical and organizational implementation	Respect or challenge existing technical and organizational infrastructures—and be prepared to take the consequences (56)	Move secure prototypes to a living laboratory (53) setting for further design, development, and test before rollout

### Challenge 1: Developing a Proper Understanding of Users' Practices and Needs

Stroke caregivers often demand to be involved in the decision-making process in care to ensure the practices implemented consider the survivors and their individual needs (39). While user-centered design allows for a clear understanding of user needs from different groups of users including primary, secondary, and some tertiary users (29), conducting research with older adults could be challenging (59) as the average age of stroke caregivers is expected to be >55 years (60). However, Wilkinson and Cornish (61) argue that user-centered design, especially PD approach, could be used to involve the real-world users in the design and development process as it ensures tools promote increased participation irrespective of the age.

PD draws from ethnographically inspired fieldwork (i.e., interviews, observations, workshops, thinking aloud, and so on) during a normal workday of the user (62) to gain firsthand experiences with current work practices (57). Through an understanding of current work practices, researchers can form design engagements according to local needs and respond to issues defined by the intended user within the community (63). A primary concern in PD is that it consists of the distribution of power, making it difficult to utilize technology to meet the needs of the intended user (56). Hence, much of the existing body of research considers the inclusion of Computer-supported cooperative work to limit unforeseen tensions and ensure researchers shape the collaborative design engagements to align with a diverse group of users' needs and practices (63), while being cautious about expecting one group to deliver valuable data without getting valuable feedback (58).

### Challenge 2: Codesign of Innovative and Sustainable Solutions

In user-centered design, once the user needs and requirements are identified, a process of design, evaluate, and reiterate is carried out. This iterative process refines a software prototype based on a collaboration between intended users and the researchers to eventually better support the intended user (i.e., caregiver) in their daily activities (64). Moreover, it allows for the researcher to identify possible usability errors that may impact the users' ability to interact with the system (65) during recovery and care of the person living with stroke.

The PD process during codesign relies on two principal values, participation and democracy, to involve a range of individuals with diversity in experiences and knowledge (66, 67). These principal values are expected to be maintained throughout the design process, thereby enabling trust and facilitating mutual learning and commitment toward developing a system that meets the needs of the intended user (50). One way to practice these values is by facilitating a variety of workshops, storyboards, mock-ups, probes, scenarios, walk-throughs, games, collaborative prototyping, etc. (66, 68, 69). These are to ensure equal collaboration in the design of innovative and sustainable solutions based on individual knowledge and perspectives (70).

### Challenge 3: Technical and Organizational Implementation

The design of any technology in healthcare should focus both on technology and healthcare outcomes. Past healthcare literature focuses on only one aspect (i.e., health or technology) (55), which has raised some concerns in the past regarding its sustainability or adherence over extended periods. Hence, to create sustainable solutions for stroke caregivers, it is necessary for the system to meet the visions of the different stakeholders involved in the care (10), such as caregivers, survivors, medical professionals, rehabilitation specialists, etc. This is typically achieved through multiple iterations where the goals, needs, and potentials are constantly evaluated, leading to the formation of successful systems for recovery and care (71). According to Schuurman and De Marez (72) and Andersen et al. (73), it is possible to perform such practices through the use of living laboratories. The living laboratory is a concept that encompasses diverse concepts driven by local innovation activities stated by different stakeholders to improve their everyday lives (74). In general, living laboratories include codesign test beds, collaboration, and knowledge management tools to support interaction between multiple stakeholders, communities, and organizations (75) to create sustainable technological solutions that improve everyday life (74, 75), therefore allowing for the researcher to identify issues related to the technical and organizational implementation while being prepared to manage its consequences (56).

## Conclusions and Future Research

In conclusion, there are several issues highlighted in the stroke caregiving technology literature that need to be addressed to promote better success, long-term acceptance, and engagement of the designed solutions. To achieve these goals, future research in stroke caregiving technology needs to focus more on improving user participation in the design and development through proper understanding of the user practices and needs, inclusion of codesign solutions, and technical and organizational implementation. These have been demonstrated in the literature considering PD and computer-supported collaborative work approaches.

## Data Availability Statement

The original contributions presented in the study are included in the article/supplementary material, further inquiries can be directed to the corresponding author/s.

## Author Contributions

EL initiated this study to address the methodological issues in stroke caregiving technology through past literature studies in healthcare intervention design. EL performed this study under the supervision of FK, who provided critical comments that enabled a clear presentation of findings within the final manuscript. Further, EL drafted the manuscript that was revised by FK, MA, AF, LR, PL, and JG. All authors contributed to the article and approved the submitted version.

## Conflict of Interest

The authors declare that the research was conducted in the absence of any commercial or financial relationships that could be construed as a potential conflict of interest.

## Abbreviations

mHealth: mobile health
App: application
PD: participatory design
CSCW: computer-supported cooperative work.
